# Supplementary material for: Parasites of Free-Ranging and Captive American Primates: A Systematic Review
Source: Microorganisms. 2021 Dec 9;9(12):2546. doi: 10.3390/microorganisms9122546 (PMC8706906; doi:10.3390/microorganisms9122546)
Supplement: Supplementary file 1 [file microorganisms-09-02546-s001.zip › Supplementary File S1.pdf]

Supplementary File S1: parasite – host list

Parasites of free-ranging and captive Neotropical primates: A systematic review

Parasite - host list

Including reports from molecular and morphological diagnostic methods, and excluding others (Serology, ELISA, indirect ELISA, indirect agglutination assays, western blood IgG assays, immunochromatographic assays, sero K-SeT rapid diagnostic tests, indirect immunofluorescence assays, immunohistochemical assays, antigen-based rapid diagnostic tests, TESA-blot).

Endoparasites

PROTOZOA Owen, 1858

Family Balantidiidae Doflein and Reichenow, 1929

Balantidium sp. Claparède & Lachmann, 1858

Alouatta palliata, Ecuador [1]

Balantidium coli Stein, 1863

Alouatta seniculus, Colombia* [2]

*Records from captive primates.

Balantioides sp. Alexeieff, 1931

Aotus nigriceps, Peru [3]

Family Blastocystidae Jiang and He, 1993

Blastocystis sp. Jaekel, 1918

Alouatta sp., Colombia [4]

Alouatta caraya, Brazil* [5]

Alouatta caraya, Argentina* [6]

Alouatta guariba, Brazil* [5]

Alouatta palliata, Mexico [7]

Alouatta palliata, Ecuador [1]

Alouatta pigra, Mexico [7]

Alouatta seniculus, Brazil* [5]

Ateles belzebuth, Brazil* [5]

Ateles fusciceps, Brazil* [5]

Ateles paniscus, China* [8]

Callithrix jacchus, Brazil* [5]

Lagothrix lagotricha, Brazil* [5]

Leontopithecus chrysomelas, Brazil* [5]

Mico argentatus, Brazil* [5]

Saimiri sciureus, China* [8]

Sapajus apella, Brazil* [5]

*Records from captive primates.

Blastocystis hominis Brumpt, 1912

Alouatta caraya, Argentina* [6]

Alouatta caraya, Argentina [6]

*Records from captive primates.

Family Cryptosporidiidae Léger 1911

Cryptosporidium sp. Tyzzer, 1907

Alouatta caraya, Argentina* [6]

Callicebus nigrifrons, Brazil* [9]

Lagothrix flavicauda, Peru [10]

*Records from captive primates.

Cryptosporidium parvum Tyzzer, 1912

Ateles paniscus, Brazil* [11]

Callithrix sp., Brazil* [11]

*Records from captive primates.

Family Eimeriidae Minchin, 1903

Cyclospora sp. Schneider, 1881

Alouatta palliata, Ecuador [1]

Eimeria sp. Schneider, 1875

Alouatta caraya, Argentina [12]

Isospora sp. Schneider, 1881

Alouatta palliata, Ecuador [1]

Family Endolimacidae Cavalier-Smith, 2004

Endolimax sp. Kuenen and Swellengrebel, 1913

Saguinus leucopus, Colombia [13]

Endolimax nana Wenyon and O'connor, 1917

Alouatta seniculus, Colombia* [2]

*Records from captive primates.

Iodamoeba sp. Dobell, 1919

Alouatta palliata, Ecuador [1]

Family Entamoebidae Chatton, 1925

Lagothrix lagotricha, Colombia [14]

Lagothrix lagotricha, Colombia* [14]

*Records from captive primates.

Entamoeba sp. Casagradi and Barbagallo, 1895

Alouatta palliata, Ecuador [1]

Alouatta palliata, Mexico [15]

Alouatta pigra, Mexico [15]

Aotus nigriceps, Peru [3]

Saguinus leucopus, Colombia [13]

Entamoeba coli Grasii, 1879

Alouatta caraya, Argentina* [12]

Alouatta caraya, Argentina [12]

Alouatta caraya, Argentina [6]

Alouatta pigra, Mexico [16]

Alouatta seniculus, Colombia* [2]

Aotus sp., Colombia* [2]

Cebus albifrons, Colombia* [2]

Lagothrix flavicauda, Peru [10]

*Records from captive primates.

Entamoeba histolytica Schaudinn, 1903

Alouatta seniculus, Colombia* [2]

Aotus sp., Colombia* [2]

Cebus albifrons, Colombia* [2]

*Records from captive primates.

Entamoeba histolytica/dispar/moskovskii/nuttalli

Cebus albifrons, Ecuador* [17]

*Records from captive primates.

Entamoeba sp. / *Endolimax* sp.

Alouatta palliata, Costa Rica [18]

Family Giardiidae Kulda and Nohýnková, 1978

Giardia sp. Ancey, 1910

Alouatta caraya, Brazil* [19]

Alouatta guariba, Brazil* [19]

Alouatta palliata, Costa Rica [20]

Aotus trivirgatus, Brazil* [19]

Ateles sp., Brazil* [19]

Brachyteles arachnoides, Brazil* [19]

Callithrix penicillata, Brazil* [19]

Cebus kaapori, Brazil* [19]

Lagothrix lagotricha, Brazil* [19]

Leontopithecus chrysopygus, Brazil* [19]

Leontopithecus rosalia, Brazil* [19]

Saimiri sciureus, Colombia* [21]

*Records from captive primates.

Giardia duodenalis Kulda and Nohýnková, 1995

Alouatta caraya, Argentina [22]

Giardia lamblia Kulda and Nohýnková, 1995

Alouatta caraya, Argentina* [12]

Alouatta caraya, Argentina [12]

Alouatta caraya, Argentina* [6]

*Records from captive primates.

Family Dientamoebidae Grasse, 1953

Dientamoeba sp. Jepps & Dobell, 1918

Alouatta palliata, Ecuador [1]

Family Plasmodiidae Mesnil, 1903

Plasmodium sp. Marchiafava and Celli, 1885

Alouatta guariba, Brazil [23]

Callithrix jacchus, Brazil* [24]

Callithrix jacchus, Brazil* [25]

Sapajus sp., Brazil* [24]

Sapajus sp., Brazil* [25]

Sapajus flavius, Brazil [26]

*Records from captive primates.

Plasmodium brasilianum Gonder and von Berenberg-Gossler, 1908

Alouatta belzebul, Brazil [27]

Alouatta caraya, Brazil [27]

Alouatta guariba, Brazil [27]

Alouatta guariba, Brazil [23]

Alouatta seniculus, Brazil [27]

Ateles chamek, Brazil [27]

Ateles paniscus, Brazil [27]

Cacajao calvus, Brazil [27]

Callithrix geoffroyi, Brazil* [28]

Cheracebus torquatus, Brazil [27]

Chiropotes albinasus, Brazil [27]

Chiropotes chiropotes, Brazil [27]

Chiropotes satanas, Brazil [27]

Lagothrix cana, Brazil [27]

Lagothrix lagotricha, Brazil [27]

Leontopithecus chrysomelas, Brazil* [28]

Leontopithecus rosalia, Brazil* [28]

Mico humeralifer, Brazil* [28]

Pithecia monachus, Brazil [27]

Pithecia pithecia, Brazil [27]

Plecturocebus brunneus, Brazil [27]

Plecturocebus moloch, Brazil [27]

Saguinus fuscicollis, Peru [29]

Saguinus imperator, Peru [29]

Saguinus martinsi, Brazil* [28]

Saguinus midas, Brazil [27]

Saimiri boliviensis, Brazil [27]

Saimiri sciureus, Brazil [27]

Saimiri ustus, Brazil [27]

Sapajus apella, Brazil [27]

Sapajus macrocephalus, Brazil [27]

*Records from captive primates.

Plasmodium falciparum Welch 1897

Alouatta seniculus, Colombia [30]

*Records from captive primates.

Plasmodium malariae Feletti and Grassi, 1889

Aotus infulatus, Brazil* [25]

Callithrix jacchus, Brazil* [25]

Chiropotes satanas, Brazil* [25]

Saguinus midas, Brazil* [25]

Sapajus sp., Brazil [25]

*Records from captive primates.

Plasmodium malariae/brasilianum

Alouatta guariba, Brazil [23]

Alouatta guariba, Brazil [31]

Alouatta seniculus, Colombia [30]

Aotus griseimembra, Colombia [30]

Ateles hybridus, Colombia [30]

Cebus versicolor, Colombia [30]

Sapajus sp., Brazil* [24]

Sapajus sp., Brazil [24]

*Records from captive primates.

Plasmodium simium Da Fonseca 1951

Alouatta guariba, Brazil [23]

Alouatta guariba, Brazil [32]

Alouatta guariba, Brazil* [33]

Alouatta guariba, Brazil* [34]

Brachyteles arachnoides, Brazil [27]

*Records from captive primates.

Plasmodium vivax/simium

Alouatta sp., Brazil [35]

Alouatta guariba, Brazil [31]

Alouatta seniculus, Colombia [30]

Ateles hybridus, Colombia [30]

Cebus versicolor, Colombia [30]

Family Chilomastigidae Cavalier-Smith 2013

Chilomastix sp. Alexeieff, 1910

Alouatta palliata, Ecuador [1]

Family Sarcocystidae Poche, 1913

Toxoplasma sp. Nicolle and Manceaux, 1909

Alouatta sp., Brazil* [36]

Saimiri sciureus, South Korea* [37]

Saimiri sciureus, Japan* [38]

*Records from captive primates.

Toxoplasma gondii Nicolle and Manceaux, 1908

Alouatta sp., Brazil* [36]

Saimiri sciureus, Colombia* [21]

Saimiri sciureus, South Korea* [37]

Saimiri sciureus, Japan* [38]

*Records from captive primates.

Family Trichomonadidae Chalmers and Pekkola, 1918

Tetratrichomonas sp. Parisi, 1910

Alouatta sara, Brazil* [39]

Callithrix penicillata, Brazil* [39]

Sapajus apella, Brazil* [39]

*Records from captive primates.

Trichomonas sp. Donné, 1836

Alouatta palliata, Costa Rica [20]

Family Trypanosomatidae Ross, 1903

Alouatta seniculus, Peru* [40]

Alouatta seniculus, Peru [40]

Aotus sp., Peru* [40]

Ateles belzebuth, Peru* [40]

Ateles chamek, Peru [40]

Cacajao calvus, Peru [40]

Cebus albifrons, Peru [40]

Lagothrix cana, Peru* [40]

Lagothrix lagotricha, Peru* [40]

Lagothrix poeppigii, Peru* [40]

Pithecia monachus, Peru [40]

Plecturocebus cupreus, Peru [40]

Saimiri boliviensis, Peru* [40]

Saimiri boliviensis, Peru [40]

Saimiri sciureus, Peru* [40]

Saimiri sciureus, Peru [40]

Sapajus macrocephalus, Peru* [40]

Sapajus macrocephalus, Peru [40]

*Records from captive primates

Leishmania sp. Ross, 1903

Alouatta seniculus, French Guiana [41]

Sapajus cay, Brazil [42]

Leishmania amazonensis Lainson & Shaw, 1972

Alouatta caraya, Argentina [43]

Leishmania braziliensis Vianna, 1911

Aotus trivirgatus, Panama [44]

Saguinus geoffroyi, Panama [44]

Alouatta caraya, Argentina [43]

Leishmania braziliensis/ infantum/amazonensis

Callithrix sp., Brazil [45]

Leishmania guyanensis Floch, 1954

Alouatta seniculus, French Guiana [41]

Leishmania infantum Nicolle, 1908

Alouatta caraya, Argentina [43]

Alouatta guariba, Brazil* [46]

*Records from captive primates

Leishmania shawi Lainson *et al*. 1989

Sapajus apella, Brazil [47]

Trypanosoma sp. Gruby, 1843

Callithrix geoffroyi, Brazil [48]

Saguinus leucopus, Colombia [13]

*Records from captive primates

Trypanosoma cruzi Chagas, 1909

Aotus azarae, Brazil* [49]

Ateles paniscus, Brazil* [49]

Cebus albifrons. Peru [40]

Cebus olivaceus, Brazil* [49]

Lagothrix cana, Brazil* [49]

Lagothrix poeppigii, Peru [40]

Pithecia monachus, Brazil* [49]

Plecturocebus oenanthe, Peru [40]

Saguinus midas, Brazil* [49]

Saimiri boliviensis, Peru [40]

Saimiri sciureus, Peru* [40]

Saimiri sciureus, Brazil* [49]

Saimiri sciureus, Peru [40]

Saimiri ustus, Brazil* [49]

Sapajus sp., Brazil* [49]

Sapajus apella, Brazil* [49]

Sapajus macrocephalus, Peru [40]

Sapajus macrocephalus, Peru* [40]

*Records from captive primates

Trypanosoma minasense Chagas, 1908

Callithrix geoffroyi, Brazil [48]

Callithrix penicillata, Brazil [50]

ANIMALIA Linnaeus, 1758

Phylum Nematoda Rudolphi, 1808

Saguinus leucopus, Colombia [13]

Sapajus flavius, Brazil [26]

Family Ancylostomatidae Looss, 1905

Saguinus leucopus, Colombia [13]

Ancylostoma sp. Dubini, 1843

Lagothrix flavicauda, Peru [10]

Saimiri sciureus, Colombia* [21]

*Records from captive primates.

Family Ascarididae Baird, 1853

Aotus nigriceps, Peru [3]

Lagothrix lagotricha, Colombia [14]

Ascaris sp. Linnaeus, 1758

Alouatta guariba, Brazil [51]

Saguinus leucopus, Colombia [13]

Sapajus nigritus, Argentina [52]

Ascaris lumbricoides Linnaeus, 1758

Alouatta seniculus, Colombia* [2]

*Records from captive primates.

Family Capillariidae Neveu-Lemaire, 1936

Capillaria sp. Zeder, 1800

Lagothrix flavicauda, Peru [10]

Alouatta palliata, Ecuador [1]

Cebus albifrons, Ecuador [53]

Family Filaroididae Schulz, 1951

Filariopsis sp. Van Thiel, 1926

Sapajus nigritus, Argentina [52]

Family Gongylonematidae Hall, 1916

Saguinus weddelli, Peru [54]

Saguinus imperator, Peru [54]

Gongylonema sp. Molin, 1857

Callithrix geoffroyi, Brazil [55]

Family Habronematidae Ivaschkin, 1961

Parabronema sp. Baylis, 1921

Alouatta pigra, Mexico [56]

Family Metastrongylidae Leiper, 1909

Saguinus leucopus, Colombia [13]

Family Molineidae *S*krjabin and Schulz, 1937

Molineus torulosus Molin 1861

Sapajus apella, Brazil* [57]

Sapajus flavius, Brazil* [57]

Sapajus libidinosus, Brazil* [57]

*Records from captive primates.

Family Onchocercidae Leiper, 1911

Alouatta macconnelli, French Guiana [58]

Brugia sp. Buckley, 1960

Alouatta macconnelli, French Guiana [58]

Dipetalonema freitasi Bain, Diagne & Muller, 1987

Cacajao calvus, Peru [59]

Dipetalonema gracile Rudolphi, 1809

Lagothrix poeppiigii, Peru [60]

Pithecia monachus, Peru [59]

*Saimiri sciureus, China** [61]

*Records from captive primates.

Mansonella sp. Faust, 1929

Alouatta macconnelli, French Guiana [58]

Family Oxyuridae Cobbold, 1864

Lagothrix lagotricha, Colombia [14]

Lagothrix lagotricha, Colombia* [14]

Saimiri sciureus, Colombia [21]

*Records from captive primates.

Enterobius sp. Leach, 1853

Alouatta palliata, Ecuador [1]

Enterobius vermicularis Linnaeus, 1758

Alouatta seniculus, Colombia* [2]

Cebus albifrons, Colombia* [2]

*Records from captive primates.

Trypanoxyuris sp. Vevers, 1923

Alouatta guariba, Brazil [51]

Alouatta palliata, Costa Rica [18]

Alouatta palliata, Ecuador [1]

Alouatta pigra, Mexico [62]

Alouatta pigra, Mexico [56]

Aotus nigriceps, Peru [3]

Leontopithecus chrysomelas, Brazil [63]

Trypanoxyuris atelis Cameron, 1929

Ateles geoffroyi, Mexico [64]

Ateles geoffroyi, Mexico [65]

Ateles geoffroyi, Mexico* [65]

Ateles geoffroyi, Costa Rica [64]

*Records from captive primates.

Trypanoxyuris atelophora Kreis, 1932

Ateles geoffroyi, Mexico [64]

Ateles geoffroyi, Costa Rica [64]

Trypanoxyuris callithricis Solomon, 1933

Callithrix sp., Brazil [66]

Trypanoxyuris kemuimae Solórzano-García, Link Ospina, Rondón & Pérez-Ponce de León, 2020

Alouatta seniculus, Colombia [67]

Trypanoxyuris kotudoi Solórzano-García, Link Ospina, Rondón & Pérez-Ponce de León, 2020

Alouatta seniculus, Colombia [67]

Trypanoxyuris minutus Schneider, 1866

Alouatta sp., Mexico [65]

Alouatta guariba., Brazil [66]

Alouatta guariba., Brazil [68]

Alouatta palliata, Costa Rica [64]

Alouatta palliata, Mexico [64]

Alouatta palliata, Nicaragua [64]

Trypanoxyuris multilabiatus Solórzano-García *et al.*, 2016

Alouatta palliata, Costa Rica [64]

Alouatta palliata, Mexico [64]

Alouatta palliata, Nicaragua [64]

Trypanoxyuris seunimii Solórzano-García, Link Ospina, Rondón & Pérez-Ponce de León, 2020

Alouatta seniculus, Colombia [67]

Family Physalopteridae Railliet, 1893

Sapajus nigritus, Argentina [52]

Physaloptera sp. Rudolphi, 1819

Sapajus nigritus, Brazil [66]

Family Rictulariidae Railliet, 1916

Pterygodermatites nycticebi Mönnig, 1926

Callimico sp., Switzerland* [69]

*Records from captive primates.

Family Spiruridae Oerley, 1885

Leontopithecus chrysomelas, Brazil [63]

Saguinus leucopus, Colombia [13]

Family Strongylidae Baird, 1853

Aotus nigriceps, Peru [3]

Lagothrix lagotricha, Colombia [14]

Lagothrix lagotricha, Colombia* [14]

*Records from captive primates.

"Strongyles"

Cebus albifrons, Ecuador [53]

Family Strongyloididae Chitwood and McIntosh, 1934

Strongyloides sp. Grassi, 1879

Alouatta caraya, Argentina [70]

Alouatta palliata, Costa Rica [18]

Alouatta palliata, Ecuador [1]

Alouatta seniculus, Colombia* [2]

Aotus nigriceps, Peru [3]

Cebus albifrons, Ecuador [53]

Cebus albifrons, Ecuador* [53]

Lagothrix flavicauda, Peru [10]

Saguinus leucopus, Colombia [13]

Saimiri sciureus, Colombia* [21]

Sapajus nigritus, Argentina [52]

*Records from captive primates.

Strongyloides cebus Darling, 1911

Lagothrix flavicauda, Peru [10]

Strongyloides stercoralis Bavay, 1876

Pithecia monachus, Peru* [71]

*Records from captive primates.

Family Subuluridae Yorke and Maplestone, 1926

Sapajus nigritus, Argentina [52]

Primasubulura sp. Inglis, 1958

Callithrix sp., Brazil [66]

Leontopithecus chrysomelas., Brazil [63]

Saguinus imperator, Peru [54]

Saguinus weddelli, Peru [54]

Family Trichinellidae Ward, 1907

Lagothrix lagotricha, Colombia* [14]

*Records from captive primates.

Family Trichostrongylidae Leiper 1912

Lagothrix lagotricha, Colombia* [14]

*Records from captive primates.

Trichostrongylus sp. Looss, 1905

Saguinus leucopus, Colombia [13]

Family Trichuridae Railliet, 1915

Trichuris sp. Roederer, 1761

Sapajus nigritus, Argentina [52]

ANIMALIA Linnaeus, 1758

Phylum Acanthocephala Kohlreuther, 1771

Family Oligacanthorhynchidae Southwell and Macfie, 1925

Pachysentis sp. Meyer, 1931

Alouatta guariba, Brazil [66]

Callithrix sp., Brazil [66]

Prosthenorchis sp. Travassos, 1915

Leontopithecus chrysomelas, Brazil [66]

Leontopithecus chrysomelas, Brazil [63]

Saguinus imperator, Peru [54]

Saguinus leucopus, Colombia [13]

Saguinus weddelli, Peru [54]

Sapajus nigritus, Brazil [66]

Prosthenorchis elegans Diesing, 1851

Callithrix geoffroyi, Brazil [72]

Cebus albifrons, Ecuador* [53]

Lagothrix flavicauda, Peru [10]

Saimiri sciureus, Colombia* [21]

*Records from captive primates.

ANIMALIA Linnaeus, 1758

Phylum Platyhelminthes Minot, 1876

Class Cestoda Rudolphi, 1808

Lagothrix lagotricha, Colombia [14]

Leontopithecus rosalia, Brazil [66]

Saguinus imperator, Peru [54]

Saguinus weddelli, Peru [54]

Family Anoplocephalidae Cholodkovsky, 1902

Bertiella sp. Stiles and Hassall, 1902

Alouatta caraya, Paraguay [73]

Alouatta caraya, Argentina* [6]

Alouatta guariba, Brazil [66]

Alouatta guariba, Brazil [51]

*Records from captive primates.

Bertiella mucronata Meyner, 1895

Alouatta caraya, Argentina [70]

Alouatta caraya, Argentina* [70]

*Records from captive primates.

Paratriotaenia oedipomidatis Linn, 1758

Lagothrix flavicauda, Peru [10]

Family Hymenolepididae Ariola, 1899

Sapajus nigritus, Argentina [52]

Hymenolepis sp. Kaulfuss, 1824

Cebus albifrons, Ecuador [53]

Family Mesocestoididae Fuhrmann, 1907

Mesocestoides sp. Vaillant, 1863

Saguinus midas, Italy* [74]

*Records from captive primates.

ANIMALIA Linnaeus, 1758

Phylum Platyhelminthes Minot, 1876

Class Trematoda Rudolphi, 1808

Alouatta pigra, Mexico [56]

Aotus nigriceps, Peru [3]

Sapajus nigritus, Argentina [52]

Family Dicrocoeliidae Odhner, 1910

Alouatta pigra, Mexico [62]

Saguinus imperator, Peru [54]

Saguinus weddelli, Peru [54]

Controrchis sp. Price, 1928

Alouatta palliata, Costa Rica [18]

Alouatta palliata, Ecuador [1]

Alouatta pigra, Mexico [62]

Controrchis biliophilus Price, 1928

Alouatta pigra, Mexico [56]

Platynosomum sp. Looss, 1907

Callithrix sp., Brazil [66]

Callithrix geoffroyi, Brazil [55]

Sapajus nigritus, Brazil [66]

Platynosomum illiciens Braun, 1901

Callithrix penicillata, Brazil* [75]

Callithrix penicillata, Brazil* [76]

*Records from captive primates.

Ectoparasites

ANIMALIA Linnaeus, 1758

Family Ixodidae Dugès, 1834

Amblyomma aureolatum Pallas, 1772

Alouatta guariba, Brazil* [77]

*Records from captive primates.

Amblyomma dubitatum Neumann, 1899

Alouatta guariba, Brazil* [77]

*Records from captive primates.

Amblyomma parkeri Fonseca & Aragao, 1952

Alouatta guariba, Brazil* [77]

Callicebus nigrifrons, Brazil* [77]

*Records from captive primates.

Amblyomma romarioi Martins, Luz & Labruna, 2019

Callicebus nigrifrons, Brazil* [78]

*Records from captive primates.

Amblyomma sculptum Berlese, 1888

Alouatta guariba, Brazil [79]

Alouatta guariba, Brazil* [77]

*Records from captive primates.

Rhipicephalus microplus Canestrini, 1888

Alouatta seniculus, Brazil [80]

Family Oestridae Leach, 1815

Cuterebra baeri Shannon and Greenem, 1926

Aotus nancymaae, Colombia [81]

Aotus vociferans, Colombia [81]

Family Trichodectidae Kellogg, 1896

Cebidicola semiarmatus Neumann, 1913

Alouatta guariba, Brazil [82]

Family Hectopsyllidae Baker, 1904

Tunga penetrans Linnaeus, 1758

Alouatta guariba, Brazil [51]

References

1. Helenbrook, W.D.; Stehman, S. V.; Shields, W.M.; Whipps, C.M. Association of Anthropogenic Disturbances and Intestinal Parasitism in Ecuadorian Mantled Howler Monkeys, *Alouatta palliata aequatorialis*. *Folia Primatol.* **2017**, *88*, 307–322, doi:10.1159/000479687.

2. de la Hoz, D.M.E.; Cañate González, A.S.; Vergel, E.F.; Payares Ramírez, K.J.; Morales López, S.E. Parasitic and fungal agents in *Aotus* sp., *Alouatta seniculus* and *Cebus albifrons* in the Colombian Caribbean. *Rev. Investig. Vet. del Peru* **2020**, *31*, doi:10.15381/RIVEP.V31I4.17632.

3. Helenbrook, W.D.; Nelson, A.; Paras, K.L.; Solorzano-Garcia, B. Intestinal Parasitism in Free-Ranging Black-Headed Night Monkeys, *Aotus nigriceps*, of Southeastern Peru. *Int. J. Primatol.* **2020**, *41*, 458–470, doi:10.1007/s10764-020-00146-7.

4. Ramírez, J.D.; Sánchez, L.V.; Bautista, D.C.; Corredor, A.F.; Flórez, A.C.; Stensvold, C.R. *Blastocystis* subtypes detected in humans and animals from Colombia. *Infect. Genet. Evol.* **2014**, *22*, 223–228, doi:10.1016/j.meegid.2013.07.020.

5. Oliveira-Arbex, A.P.; David, É.B.; Tenório, M. da S.; Cicchi, P.J.P.; Patti, M.; Coradi, S.T.; Lucheis, S.B.; Jim, J.; Guimarães, S. Diversity of *Blastocystis* subtypes in wild mammals from a zoo and two conservation units in southeastern Brazil. *Infect. Genet. Evol.* **2020**, *78*, doi:10.1016/j.meegid.2019.104053.

6. Servián, A.; Zonta, M.L.; Cociancic, P.; Falcone, A.; Ruybal, P.; Capasso, S.; Navone, G.T. Morphological and molecular characterization of *Bertiella* sp. (Cestoda, Anoplocephalidae) infection in a human and howler monkeys in Argentina. *Parasitol. Res.* **2020**, *119*, 1291–1300, doi:10.1007/s00436-020-06615-5.

7. Villanueva-Garcia, C.; Gordillo-Chavez, E.J.; Lopez-Escamilla, E.; Rendon-Franco, E.; Muñoz-Garcia, C.I.; Gama, L.; Martinez-Flores, W.A.; Gonzalez-Rodriguez, N.; Romero-Valdovinos, M.; Diaz-Lopez, H.; et al. Clarifying the Cryptic Host Specificity of *Blastocystis* spp. isolates from *Alouatta palliata* and *A. Pigra* Howler Monkeys. *PLoS One* **2017**, *12*, 1–15, doi:10.1371/journal.pone.0169637.

8. Ma, L.; Qiao, H.; Wang, H.; Li, S.; Zhai, P.; Huang, J.; Guo, Y. Molecular prevalence and subtypes of *Blastocystis* sp. in primates in northern China. *Transbound. Emerg. Dis.* **2020**, *67*, 2789–2796, doi:10.1111/tbed.13644.

9. Bofill, I.; Pereira de Souza, S.; Dias de Paula, F.; Gennari, S.; Quilez, J. *Cryptosporidium* spp. en *Callicebus nigrifons*: reporte de un caso de diarrea aguda en un centro de rescate de primates del estado de São Paulo, Brasil. *Neotrop. Primates* **2018**, *24*, 72–75.

10. Reátegui Guzmán, E.H.; Piperis, R.E.; Cornejo Fernandez, F.M.; Quispe Huacho, M.A.; Tantaleán Vidaurre, M.E. Gastrointestinal parasites in wild yellow-tailed woolly monkey (*Lagothrix flavicauda*) in the Corosha district, Amazonas, Peru. *Rev. Investig. Vet. del Peru* **2020**, *31*, doi:10.15381/RIVEP.V31I4.19030.

11. Snak, A.; Da Silveira Delgado, L.E.; Osaki, S.C. *Cryptosporidium parvum* in captive primates of Parque Municipal Danilo Galafassi, Paraná, Brazil. *Semin. Agrar.* **2019**, *40*, 987–992, doi:10.5433/1679-0359.2019v40n2p987.

12. Milozzi, C.; Bruno, G.; Cundom, E.; Mudry, M.D.; Navone, G.T. Intestinal parasites of *Alouatta caraya* (Primates, Ceboidea): preliminary study in semi-captivity and in the wild in Argentina. *Mastozoología Neotrop.* **2012**, *19*, 271–278.

13. Acevedo-Garcés, Y.; Álvarez-Cardona, J.; Vargas-Valencia, V.; Hernández-Castro, C.; García-Montoya, G.; Soto-Calderón, I. Clinical and parasitological evaluation of White-footed Tamarins (Primates-Cebidae- *Saguinus leucopus*) from two free-range populations located in San Carlos and San Rafael (Antioquia, Colombia). *Rev. CES Med. Vet. y Zootec.* **2014**, *9*.

14. Quiroga-González, C.; Jiménez, E.; Galvis, N.F.; Ramírez, M.A.; Ortiz, M.; González, C.; Stevenson, P.R. First records of gastrointestinal parasites in woolly monkeys (*Lagothrix lagotricha*) in Colombia, from wild, captive and reintroduced individuals. *Neotrop. Primates* **2019**, *25*, 38–43.

15. Villanueva-García, C.; Gordillo-Chávez, E.J.; Baños-Ojeda, C.; Rendón-Franco, E.; Muñoz-García, C.I.; Carrero, J.C.; Córdoba-Aguilar, A.; Maravilla, P.; Galian, J.; Martínez-Hernández, F.; et al. New Entamoeba group in howler monkeys (*Alouatta* spp.) associated with parasites of reptiles. *Parasitol. Res.* **2017**, *116*, 2341–2346, doi:10.1007/s00436-017-5519-6.

16. Martínez-Mota, R.; Garber, P.A.; Palme, R.; Gillespie, T.R. The relative effects of reproductive condition, stress, and seasonality on patterns of parasitism in wild female black howler monkeys (*Alouatta pigra*). *Am. J. Primatol.* **2017**, *79*, doi:10.1002/ajp.22669.

17. Martin-Solano, S.; Carrillo-Bilbao, G.A.; Ramirez, W.; Celi-Erazo, M.; Huynen, M.C.; Levecke, B.; Benitez-Ortiz, W.; Losson, B. Gastrointestinal parasites in captive and free-ranging *Cebus albifrons* in the Western Amazon, Ecuador. *Int. J. Parasitol. Parasites Wildl.* **2017**, *6*, 209–218, doi:10.1016/j.ijppaw.2017.06.004.

18. Chinchilla Carmona, M.; Guerrero Bermúdez, O.; Gutiérrez-Espeleta, G.; Sánchez Porras, R.; Rodríguez Ortiz, B. Parásitos intestinales en monos congo *Alouatta palliata* (Primates: Cebidae) de Costa Rica. *Rev. Biol. Trop.* **2005**, *53*, 3–4.

19. Chagas, C.R.F.; Gonzalez, I.H.L.; Salgado, P.A.B.; Rodrigues, B.; Ramos, P.L. *Giardia* spp., ten years of parasitological data in the biggest zoo of Latin America. *Ann. Parasitol.* **2019**, *65*, 35–51, doi:10.17420/ap6501.181.

20. Chinchilla, M.; Guerrero, O.; Gutiérrez-Espeleta, G.; Porras, R.; Ortiz, B. Parásitos intestinales en monos congo *Alouatta palliata* (Primates: Cebidae) de Costa Rica. *Rev. Biol. Trop.* **2005**, *53*, 437–445.

21. Botero, L.; Fernández, A.; Forero, A.; Rosas, S.; Soler-Tovar, S. Análisis retrospectivo de las enfermedades parasitarias del mono ardilla (*Saimiri sciureus*) en dos condiciones ex situ en el noroccidente de los Andes suramericanos. *Rev. Med. Vet. (Bogota).* **2011**, *22*, 85–93.

22. Kuthyar, S.; Kowalewski, M.M.; Roellig, D.M.; Mallott, E.K.; Zeng, Y.; Gillespie, T.R.; Amato, K.R. Effects of anthropogenic habitat disturbance and *Giardia duodenalis* infection on a sentinel species' gut bacteria. *Ecol. Evol.* **2021**, *11*, 45–57, doi:10.1002/ece3.6910.

23. Abreu, F.V.S. de; Santos, E. Dos; Mello, A.R.L.; Gomes, L.R.; Alvarenga, D.A.M. de; Gomes, M.Q.; Vargas, W.P.; Bianco-Júnior, C.; Pina-Costa, A. de; Teixeira, D.S.; et al. Howler monkeys are the reservoir of malarial parasites causing zoonotic infections in the Atlantic forest of Rio de Janeiro. *PLoS Negl. Trop. Dis.* **2019**, *13*, e0007906, doi:10.1371/journal.pntd.0007906.

24. Figueiredo, M.A.P.; Di Santi, S.M.; Manrique, W.G.; André, M.R.; Machado, R.Z. Serological and molecular techniques applied for identification of *Plasmodium* spp. in blood samples from non human primates. *Rev. Bras. Parasitol. Vet.* **2018**, *27*, 363–376, doi:10.1590/s1984-296120180043.

25. Figueiredo, M.; Di Santi, S.; Gómez, W.; André, M.; Zacarias, R. Identification of *Plasmodium* spp . in Neotropical primates of Maranhense Amazon in Northeast Brazil. *PLoS One* **2017**, *12*, 1–14.

26. Bueno, M.G.; Catão-Dias, J.L.; de Oliveira Laroque, P.; Arruda Vasconcellos, S.; Ferreira Neto, J.S.; Gennari, S.M.; Ferreira, F.; Laurenti, M.D.; Umezawa, E.S.; Kesper, N.; et al. Infectious Diseases in Free-Ranging Blonde Capuchins, *Sapajus flavius*, in Brazil. *Int. J. Primatol.* **2017**, *38*, 1017–1031, doi:10.1007/s10764-017-9994-5.

27. Deane, L.M. Simian malaria in Brazil. *Mem Inst Oswaldo Cruz, Rio Janeiro* **1992**, *87*, 1–20.

28. Alvarenga, D.A.M.; Pina-Costa, A.; Bianco, C.; Moreira, S.B.; Brasil, P.; Pissinatti, A.; Daniel-Ribeiro, C.T.; Brito, C.F.A. New potential *Plasmodium brasilianum* hosts: Tamarin and marmoset monkeys (family

Callitrichidae). *Malar. J.* **2017**, *16*, 1–7, doi:10.1186/s12936-017-1724-0.

29. Erkenswick, G.A.; Watsa, M.; Pacheco, M.A.; Escalante, A.A.; Parker, P.G. Chronic *Plasmodium brasilianum* infections in wild Peruvian tamarins. *PLoS One* **2017**, *12*, doi:10.1371/journal.pone.0184504.

30. Rondón, S.; León, C.; Link, A.; González, C. Prevalence of *Plasmodium* parasites in non-human primates and mosquitoes in areas with different degrees of fragmentation in Colombia. *Malar. J.* **2019**, *18*, 1–10, doi:10.1186/s12936-019-2910-z.

31. De Abreu, F.V.S.; Gomes, L.R.; Mello, A.R.L.; Bianco-Júnior, C.; De Pina-Costa, A.; Dos Santos, E.; Teixeira, D.S.; Brasil, P.; Daniel-Ribeiro, C.T.; Lourenço-De-Oliveira, R.; et al. Frozen blood clots can be used for the diagnosis of distinct *Plasmodium* species in man and non-human primates from the Brazilian Atlantic Forest. *Malar. J.* **2018**, *17*, 1–5, doi:10.1186/s12936-018-2485-0.

32. Brasil, P.; Zalis, M.G.; de Pina-Costa, A.; Siqueira, A.M.; Júnior, C.B.; Silva, S.; Areas, A.L.L.; Pelajo-Machado, M.; de Alvarenga, D.A.M.; da Silva Santelli, A.C.F.; et al. Outbreak of human malaria caused by *Plasmodium simium* in the Atlantic Forest in Rio de Janeiro: a molecular epidemiological investigation. *Lancet Glob. Heal.* **2017**, *5*, e1038–e1046, doi:10.1016/S2214-109X(17)30333-9.

33. De Alvarenga, D.A.M.; Culleton, R.; De Pina-Costa, A.; Rodrigues, D.F.; Bianco, C.; Silva, S.; Nunes, A.J.D.; De Souza, J.C.; Hirano, Z.M.B.; Moreira, S.B.; et al. An assay for the identification of *Plasmodium simium* infection for diagnosis of zoonotic malaria in the Brazilian Atlantic Forest. *Sci. Rep.* **2018**, *8*, doi:10.1038/s41598-017-18216-x.

34. da Fonseca, F. *Plasmodium* of a primate of Brazil. *Mem. Inst. Oswaldo Cruz* **1951**, *49*.

35. Buery, J.C.; Rodrigues, P.T.; Natal, L.; Salla, L.C.; Loss, A.C.; Vicente, C.R.; Rezende, H.R.; Duarte, A.M.R.D.C.; Fux, B.; Malafronte, R.D.S.; et al. Mitochondrial genome of *Plasmodium vivax/simium* detected in an endemic region for malaria in the Atlantic Forest of Espírito Santo state, Brazil: Do mosquitoes, simians and humans harbour the same parasite? *Malar. J.* **2017**, *16*, doi:10.1186/s12936-017-2080-9.

36. Santana, C.H.; de Oliveira, A.R.; dos Santos, D.O.; Pimentel, S.P.; de Souza, L. dos R.; Moreira, L.G.A.; Braz, H.M.B.; de Carvalho, T.P.; Lopes, C.E.B.; Oliveira, J.B.S.; et al. Genotyping of *Toxoplasma gondii* in a lethal toxoplasmosis outbreak affecting captive howler monkeys (*Alouatta* sp.). *J. Med. Primatol.* **2020**, doi:10.1111/jmp.12506.

37. Oh, H.; Eo, K.Y.; Gumber, S.; Hong, J.J.; Kim, C.Y.; Lee, H.H.; Jung, Y.M.; Kim, J.; Whang, G.W.; Lee, J.M.; et al. An outbreak of toxoplasmosis in squirrel monkeys (*Saimiri sciureus*) in South Korea. *J. Med. Primatol.* **2018**, *47*, 238–246, doi:10.1111/jmp.12344.

38. Nishimura, M.; Goyama, T.; Tomikawa, S.; Fereig, R.M.; El-Alfy, E.S.N.; Nagamune, K.; Kobayashi, Y.; Nishikawa, Y. Outbreak of toxoplasmosis in four squirrel monkeys (*Saimiri sciureus*) in Japan. *Parasitol. Int.* **2019**, *68*, 79–86, doi:10.1016/j.parint.2018.10.008.

39. dos Santos, C.S.; de Jesus, V.L.T.; McIntosh, D.; Carreiro, C.C.; Batista, L.C.O.; do Bomfim Lopes, B.; Neves, D.M.; Lopes, C.W.G. Morphological, ultrastructural, and molecular characterization of intestinal tetratrichomonads isolated from non-human primates in southeastern Brazil. *Parasitol. Res.* **2017**, *116*, 2479–2488, doi:10.1007/s00436-017-5552-5.

40. Aysanoa, E.; Mayor, P.; Mendoza, A.P.; Zariquiey, C.M.; Morales, E.A.; Pérez, J.G.; Bowler, M.; Ventocilla, J.A.; González, C.; Baldeviano, G.C.; et al. Molecular Epidemiology of Trypanosomatids and *Trypanosoma cruzi* in Primates from Peru. *Ecohealth* **2017**, *14*, 732–742, doi:10.1007/s10393-017-1271-8.

41. Medkour, H.; Davoust, B.; Levasseur, A.; Mediannikov, O. Molecular Evidence of *Leishmania infantum* and *Leishmania guyanensis* in Red Howler Monkey (*Alouatta seniculus*) from French Guiana. *Vector-Borne Zoonotic Dis.* **2019**, *19*, 896–900, doi:10.1089/vbz.2019.2459.

42. Porfirio, G.E. de O.; Santos, F.M.; de Macedo, G.C.; Barreto, W.T.G.; Campos, J.B.V.; Meyers, A.C.; André, M.R.; Perles, L.; de Oliveira, C.E.; Xavier, S.C. das C.; et al. Maintenance of *Trypanosoma cruzi, T. evansi* and *Leishmania* spp. by domestic dogs and wild mammals in a rural settlement in Brazil-Bolivian border. *Int. J. Parasitol. Parasites Wildl.* **2018**, *7*, 398–404, doi:10.1016/j.ijppaw.2018.10.004.

43. Martínez, M.F.; Kowalewski, M.M.; Giuliani, M.G.; Acardi, S.A.; Salomón, O.D. Molecular identification of *Leishmania* in free-ranging black and gold howler monkeys (*Alouatta caraya*) in northeastern Argentina. *Acta Trop.* **2020**, *210*, doi:10.1016/j.actatropica.2020.105534.

44. Herrer, A.; Christensen, H.; Beumer, R. Reservoir hosts of cutaneous leshmaniasis among Panamanian forest mammals. *Am. J. Trop. Med. Hyg.* **1973**, *22*.

45. Trüeb, I.; Portela, R.D.; Franke, C.R.; Carneiro, I.O.; Ribeiro, G.J.; Soares, R.P.; Barrouin-Melo, S.M. *Trypanosoma cruzi* and *Leishmania* sp. infection in wildlife from urban rainforest fragments in northeast Brazil. *J. Wildl. Dis.* **2018**, *54*, 76–84, doi:10.7589/2017-01-017.

46. Santos, R.; Oliveira, A. Leishmaniasis in non-human primates: Clinical and pathological manifestations and potential as reservoirs. *J. Med. Primatol.* 2020, *49*, 34–39.

47. R, L.; JJ, S.; RR, B.; E, S.; AA, S.; FT, S. Isolation of *Leishmania* from monkeys in the Amazon region of Brazil. *Trans Roy Soc Trop Med Hyg* **1988**, *82*.

48. Dario, M.A.; Lisboa, C.V.; Costa, L.M.; Moratelli, R.; Nascimento, M.P.; Costa, L.P.; Reis Leite, Y.L.; Llewellyn, M.S.; Das Chagas Xavier, S.C.; Rodrigues Roque, A.L.; et al. High *Trypanosoma* spp. diversity is maintained by bats and triatomines in Espírito Santo state, Brazil. *PLoS One* **2017**, *12*, doi:10.1371/journal.pone.0188412.

49. Bahia, M.; de Nazaré Leite Barros, F.; Magalhães-Matos, P.C.; de Souza Gonçalves, T.; Chiesorin Neto, L.; Oliveira Faria, D.C.L.; Aparecida Romeiro, S.; Barros Monteiro, F.O.; Góes-Cavalcante, G.; Scofield, A. *Trypanosoma cruzi* infection in captive Neotropical primates in the Brazilian Amazon. *Am. J. Primatol.* **2017**, *79*, 1–6, doi:10.1002/ajp.22590.

50. Ziccardi, M.; Lourenço-De-Oliveira, R.; Nogueira, R. The Haemoculture of *Trypanosoma minasense* Chagas, 1908; Vol. 91;.

51. Schott, D.; Ribeiro, P.R.; de Souza, V.K.; Surita, L.E.; de Amorim, D.B.; Bianchi, M.V.; Anicet, M.Z.; Alievi, M.M.; Pavarini, S.P.; de Carvalho, R.W.; et al. Clinical and pathological aspects of first report of *Tunga penetrans* infestation on southern brown howler monkey (*Alouatta guariba clamitans*) in Rio Grande do Sul, Brazil. *J. Med. Primatol.* **2020**, *49*, 315–321, doi:10.1111/jmp.12491.

52. Agostini, I.; Vanderhoeven, E.; Beldomenico, P.M.; Pfoh, R.; Notarnicola, J. First coprological survey of helminths in a wild population of black capuchin monkeys (*Sapajus nigritus*) in northeastern Argentina. *Mastozool. Neotrop.* **2018**, *25*, 269–281, doi:10.31687/SAREMMN.18.25.2.0.11.

53. Martin-Solano, S.; Carrillo-Bilbao, G.A.; Ramirez, W.; Celi-Erazo, M.; Huynen, M.C.; Levecke, B.; Benitez-Ortiz, W.; Losson, B. Gastrointestinal parasites in captive and free-ranging *Cebus albifrons* in the Western Amazon, Ecuador. *Int. J. Parasitol. Parasites Wildl.* **2017**, *6*, 209–218, doi:10.1016/j.ijppaw.2017.06.004.

54. Erkenswick, G.A.; Watsa, M.; Gozalo, A.S.; Dudaie, S.; Bailey, L.; Muranda, K.S.; Kuziez, A.; Parker, P.G. A multiyear survey of helminths from wild saddleback (*Leontocebus weddelli*) and emperor (*Saguinus imperator*) tamarins. *Am. J. Primatol.* **2019**, *81*, doi:10.1002/ajp.23063.

55. Oliveira, A.R.; Souza, T.D.; Flecher, M.C.; Gardiner, C.H.; Santos, R.L. First report of *Gongylonema* sp. in a free ranging callitrichid from the Brazilian Atlantic Forest: Case report. *Arq. Bras. Med. Vet. e Zootec.* **2019**, *71*, 777–781, doi:10.1590/1678-4162-10760.

56. Martínez-mota, R.; Gillespie, T.R.; Garber, P.A.; Palme, R. The relative effects of reproductive condition ,

stress , and seasonality on patterns of parasitism in wild female black howler monkeys (*Alouatta pigra*). **2017**, 1–12, doi:10.1002/ajp.22669.

57. Bacalhao, M.B.M.; Siqueira, R.A.S.; Nery, T.F.L.; Firmino, M. de O.; Neto, T.S. de O.; Nascimento, H.H.L.; Guerra, R.R.; Lucena, R.B. Ulcerative and granulomatous enteritis associated with *Molineus torulosus* parasitism in neotropical primates. *Pesqui. Vet. Bras.* **2016**, *36*, 1005–1008, doi:10.1590/S0100-736X2016001000014.

58. Laidoudi, Y.; Medkour, H.; Levasseur, A.; Davoust, B.; Mediannikov, O. New molecular data on filaria and its *Wolbachia* from red howler monkeys (*Alouatta macconnelli*) in French Guiana - A preliminary study. *Pathogens* **2020**, *9*, 1–23, doi:10.3390/pathogens9080626.

59. Fernandez Conga, D.; Mayor, P.; Guerreiro Giese, E.; Nascimento dos Santos, J. First report of filarial nematodes in free-living pitheciid primates. *Syst. Parasitol.* **2019**, *96*, 257–264, doi:10.1007/s11230-019-09838-y.

60. Conga, D.F.; Mayor, P.; Furtado, A.P.; Giese, E.G.; dos Santos, J.N. Occurrence of *Dipetalonema gracile* in a wild population of woolly monkey *Lagothrix poeppiigii* in the northeastern Peruvian Amazon. *Rev. Bras. Parasitol. Vet.* **2018**, *27*, 154–160, doi:10.1590/S1984-296120180014.

61. Zhang, P.; Ran, R.K.; Abdullahi, A.Y.; Shi, X.L.; Huang, Y.; Sun, Y.X.; Liu, Y.Q.; Yan, X.X.; Hang, J.X.; Fu, Y.Q.; et al. The mitochondrial genome of *Dipetalonema gracile* from a squirrel monkey in China. *J. Helminthol.* **2020**, *94*, 1–8, doi:10.1017/S0022149X18000871.

62. Martínez-Mota, R.; Pozo-Montuy, G.; Bonilla Sánchez, Y.M.; Gillespie, T.R. Effects of anthropogenic stress on the presence of parasites in a threatened population of black howler monkeys (*Alouatta pigra*). *Therya* **2018**, *9*, 161–170, doi:10.12933/therya-18-572.

63. Costa, T.S.O.; Nogueira-Filho, S.L.G.; De Vleeschouwer, K.M.; Oliveira, L.C.; de Sousa, M.B.C.; Mendl, M.; Catenacci, L.S.; Nogueira, S.S.C. Individual behavioral differences and health of golden-headed lion tamarins (*Leontopithecus chrysomelas*). *Am. J. Primatol.* **2020**, doi:10.1002/ajp.23118.

64. Solórzano García, B.; Melin, A.D.; Aureli, F.; Pérez Ponce De León, G. Unveiling patterns of genetic variation in parasite-host associations: An example with pinworms and Neotropical primates. *Parasitology* **2019**, *146*, 356–362, doi:10.1017/S0031182018001749.

65. Solórzano-García, B.; Gasca-Pineda, J.; Poulin, R.; Pérez-Ponce de León, G. Lack of genetic structure in pinworm populations from New World primates in forest fragments. *Int. J. Parasitol.* **2017**, *47*, 941–950, doi:10.1016/j.ijpara.2017.06.008.

66. Pereira, F. V.; Lucena, F.P.; Rodrigues, R.L.; Barros, L.A.; Pires, C.A.; Ferreira, A.M.R.; Mello, M.F.V. Prevalence and spatial distribution of the occurrence of helminths in free-living nonhuman primates in the State of Rio de Janeiro, Brazil. *Arq. Bras. Med. Vet. e Zootec.* **2020**, *72*, 1705–1712, doi:10.1590/1678-4162-11868.

67. Solórzano-García, B.; Ospina, A.L.; Rondón, S.; Pérez-Ponce de León, G. Pinworms of the red howler monkey (*Alouatta seniculus*) in Colombia: Gathering the pieces of the pinworm-primate puzzle. *Int. J. Parasitol. Parasites Wildl.* **2020**, *11*, 17–28, doi:10.1016/j.ijppaw.2019.11.007.

68. Barbosa, A. da S.; Dib, L.V.; Uchôa, C.M.A.; Bastos, O.M.P.; Pissinatti, A. *Trypanoxyuris* (*Trypanoxyuris*) *minutus* (Schneider, 1866) among *Alouatta guariba clamitans* (Cabrera, 1940) in the state of Rio de Janeiro, Brazil. *J. Med. Primatol.* **2017**, *46*, 101–105, doi:10.1111/jmp.12265.

69. Balsiger, A.; Federer, K.; Grimm, F.; Deplazes, P. Transmission of *Pterygodermatites nycticebi* in a colony of goeldi's monkeys (*Callimico goeldii*) and evaluation of treatment and control. *J. Zoo Wildl. Med.* **2018**, *49*, 893–901, doi:10.1638/2017-0177.1.

70. Rivero, M.R.; Feliziani, C.; De Angelo, C.; Tiranti, K.; Salomon, O.D.; Touz, M.C. *Giardia* spp., the most ubiquitous protozoan parasite in Argentina: human, animal and environmental surveys reported in the last 40 years. *Parasitol. Res.* 2020, *119*, 3181–3201.

71. Manuel Tantaleán, V.; Nofre Sánchez, P.; Perea, C.M. Natural infection by *Strongyloides stercoralis* in *Pithecia monachus* (Primates, Pitheciidae). First report in Peru. *Rev. Investig. Vet. del Peru* **2018**, *29*, 1386–1390, doi:10.15381/rivep.v29i4.15197.

72. de Oliveira, A.R.; Hiura, E.; Guião-Leite, F.L.; Flecher, M.C.; Braga, F.R.; Silva, L.P.C.; Sena, T.; de Souza, T.D. Pathological and parasitological characterization of *Prosthenorchis elegans* in a free-ranging marmoset *Callithrix geofroyi* from the Brazilian Atlantic Forest. *Pesqui. Vet. Bras.* **2017**, *37*, 1514–1518, doi:10.1590/S0100-736X2017001200025.

73. Kane, J.; Smith, R.L. *Bertiella* sp. (Meyner , 1895) infection of *Alouatta caraya* (Humbolt , 1812) in urban and natural environments in Ñeembucú , southwest Paraguay. *Am. J. Primatol.* **2020**, doi:10.1002/ajp.23166.

74. Montalbano Di Filippo, M.; Meoli, R.; Cavallero, S.; Eleni, C.; De Liberato, C.; Berrilli, F. Molecular identification of *Mesocestoides* sp. metacestodes in a captive gold-handed tamarin (*Saguinus midas*). *Infect. Genet. Evol.* **2018**, *65*, 399–405, doi:10.1016/j.meegid.2018.08.008.

75. Pinto, H.A.; Mati, V.L.T.; Pujoni, D.G.F.; Melo, A.L. *Platynosomum illiciens* (Trematoda: Dicrocoeliidae) in captive blacktufted marmoset *Callithrix penicillata* (Primates: Cebidae) from Brazil: A morphometric analyses with taxonomic comments on species of *Platynosomum* from non human primates. *J. Parasitol.* **2017**, *103*, 14–21, doi:10.1645/16-1.

76. Mati, V.L.T.; Pinto, H.A.; de Melo, A.L. Treatment of primate platynosomosis: A word of caution about the use of praziquantel in marmosets. *J. Med. Primatol.* **2021**, *50*, 60–66, doi:10.1111/jmp.12503.

77. Martins, T.F.; Milanelo, L.; Krawczak, F. da S.; Furuya, H.R.; Fitorra, L.S.; das Dores, F.T.; Pedro, V. da S.; Hippolito, A.G.; Labruna, M.B. Diversity of ticks in the wildlife screening center of São Paulo city, Brazil. *Cienc. Rural* **2017**, *47*, doi:10.1590/0103-8478cr20161052.

78. Martins, T.; Hermes, L.; Muñoz-Leal, S.; Ramirez, D.; Milanelo, L.; Marques, S.; Sanches, T.; Onofrio, V.; da C.L.Acosta, I.; Benatti, H.; et al. A new species of *Amblyomma* (Acari: Ixodidae) associated with monkeys and passerines of the Atlantic rainforest biome, Southeastern Brazil. *Ticks Tick. Borne. Dis.* **2019**, *10*, doi:10.1016/j.ttbdis.2019.07.003.

79. Martins, T.F.; Teixeira, R.H.F.; Labruna, M.B. Occurrence of ticks on wild animals received and attended at the Parque Zoológico Municipal Quinzinho de Barros, Sorocaba, São Paulo, Brazil. *Brazilian J. Vet. Res. Anim. Sci.* **2015**, *52*, 319–324, doi:10.11606/issn.1678-4456.v52i4p319-324.

80. Zimmermann, N.P.; Aguirre, A. de A.R.; Rodrigues, V. da S.; Garcia, M.V.; Medeiros, J.F.; Blecha, I.M.Z.; Duarte, P.O.; Cruz, B.C.; Cunha, R.C.; Martins, T.F.; et al. Wildlife species, Ixodid fauna and new host records for ticks in an Amazon forest area, Rondônia, Brazil. *Rev. Bras. Parasitol. Vet.* **2018**, *27*, 177–182, doi:10.1590/S1984-296120180022.

81. Roncancio, N.; Santa, M.A.; Calderón, L.M.; Gómez, E.N.; Acosta, A.; García, L.M.; Henao, B.E.; Peñuela, S.M.; Pinilla, E.A.; Poches, R.A.; et al. Differences in the prevalence of cutaneous myiasis between *Aotus vociferans* and *Aotus nancymaae* in the Colombian Amazon. *Neotrop. Primates* **2018**, *24*, 86–90.

82. Do Nascimento, R.M.; Maturano, R.; De Oliveira, M.; Daemon, E. First record of *Cebidicola semiarmatus* (Phthiraptera: Trichodectidae) on the red howler monkey, *Alouatta guariba clamintans* (primate: Atelidae) in Brazil. *Rev. Colomb. Entomol.* **2018**, *44*, 129–131, doi:10.25100/socolen.v44i1.6550.
