# Supplementary material for: Parasites of Free-Ranging and Captive American Primates: A Systematic Review
Source: Microorganisms. 2021 Dec 9;9(12):2546. doi: 10.3390/microorganisms9122546 (PMC8706906; doi:10.3390/microorganisms9122546)
Supplement: Supplementary file 1 [file microorganisms-09-02546-s001.zip › Supplementary File S2.pdf]

Supplementary File S2

Authors Declaration

To respond to the following request regarding the PRISMA Checklist (Attached):

24a Provide registration information for the review, including register name and registration number, or **state that the review was not registered.**

24b Indicate where the review protocol can be accessed, **or state that a protocol was not prepared.**

Our systematic review does not include a pre-recorded protocol. The reasons for this choice are as follows:

i) According to the PRISMA guidelines the registration of the protocol is not mandatory (http://prisma-statement.org/Protocols/Registration checklist item "Registration and protocol" n. 24 a-b")

ii) Our systematic review does not directly concern human health, therefore, the research protocol registration database suggested by the PRISMA guidelines (PROSPERO) does not allow us to proceed with the registration, pointing the following statement: "Sorry - your protocol is not eligible for inclusion in PROSPERO. We hope that this screening process has saved you the time that might be spent preparing a submission that would be rejected and that you will register future systematic reviews in PROSPERO".
